# Perspective: sleep regularity—where do we go from here?

**DOI:** 10.1093/sleep/zsag114

**Published:** 2026-04-23

**Authors:** Andrew J K Phillips, Dorothee Steven

**Affiliations:** Flinders Health and Medical Research Institute (Sleep Health), College of Medicine and Public Health, Flinders University, Bedford Park, SA, Australia; Department of Sleep and Human Factors Research, Institute for Aerospace Medicine, German Aerospace Center, Cologne, Germany

**Keywords:** biological rhythms, shift work, sleep variability, sleep consistency, intraindividual variability, circadian disruption, social jetlag

In our opinion, sleep duration has long been considered the most essential sleep index for predicting disease. Over time, this has led to a duration-centric approach to public health messaging and strategies to improve sleep health. However, this common wisdom is now being called into question. Recent findings have revealed that sleep regularity is a stronger predictor than sleep duration for several health outcomes, including mortality and cardiometabolic disease. A study in the Multi-Ethnic Study of Atherosclerosis found that higher sleep regularity was associated with lower risk on several markers of cardiometabolic disease risk, independent of sleep duration [1]. In this study, irregular sleep was associated with an increased risk to develop atherosclerotic cardiovascular disease over the next decade (partial correlation adjusting for age, sex, and race/ethnicity: *r* = −0.133, *p* < 10^−8^), whereas no such relationship was observed with total sleep time (*r* = 0.032, *p* = .16). A recent large-scale prospective study of the UK Biobank also found that higher sleep regularity was associated with a 20%–48% lower risk of all-cause mortality, being a stronger predictor than sleep duration in this cohort, which was associated with a 17%–31% lower risk; in each case, the lowest duration/regularity quintile was compared to the top four quintiles to account for potential differences in the response (e.g. U-shaped response for duration), as well as differences in the scaling of each metric [2].

In view of these discoveries, and a growing body of evidence, the US National Sleep Foundation has taken an official position on the importance of sleep regularity to health [3]. However, several limitations remain. Most studies have been cross-sectional and observational, leaving an incomplete understanding of the causal effects of sleep regularity on health, as well as the physiological mechanisms involved. Furthermore, most studies have been confounded by the fact that irregularity in one rhythm (e.g. sleep) often co-occurs with irregularity in another (e.g. light exposure [4], meal timing [5]), making it difficult to identify which one is responsible for the observed health risks. Consequently, targets for interventions are not well defined.

As research on sleep regularity continues to rapidly grow (Figure 1), this is a critical time to carefully weigh the most important unanswered questions and the most fruitful avenues for researchers to pursue. In this Perspective, we argue that there are three critical areas in need of attention: (1) understanding the drivers of irregular sleep; (2) determining the prospective risks of irregular sleep; and (3) identifying the physiological mechanisms by which irregular sleep impacts health. For each area, we formulate key unanswered research questions.

**Figure 1:**
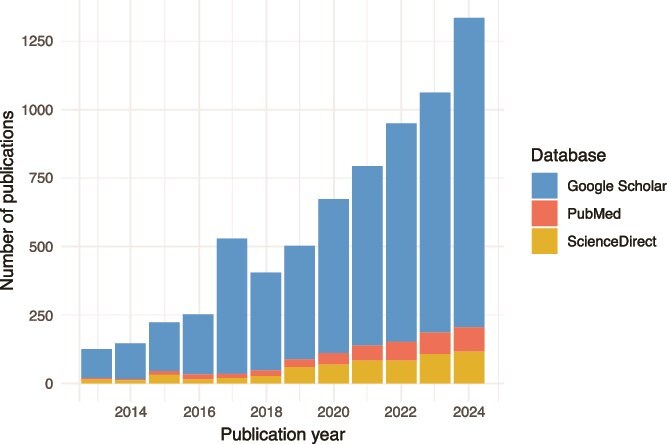
Number of published papers on sleep regularity by year and database. Search terms: “sleep regularity” OR “sleep variability” OR “sleep consistency.”

## Understanding the Drivers of Irregular Sleep

**Figure 2:**
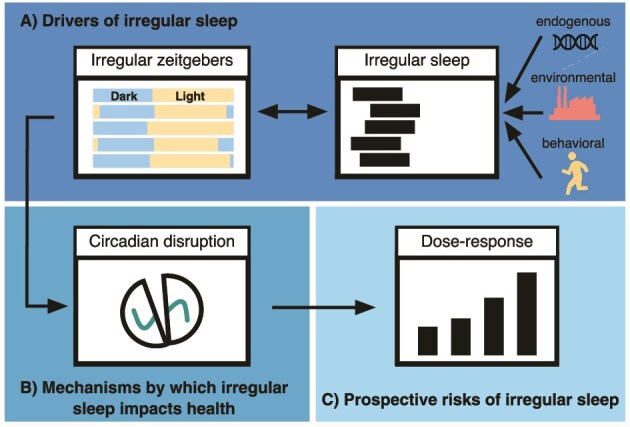
Three key areas for sleep regularity research. (A) Drivers of irregular sleep: endogenous factors (e.g. circadian response characteristics), environmental factors (e.g. social demands, shift/work schedules), and behavioral factors (e.g. exercise, eating, leisure activities) may all contribute to irregular sleep. Irregular sleep can affect and be affected by irregularity in zeitgeber rhythms (e.g. light/dark cycles), (B) mechanism by which irregular sleep impacts health: these pathways still have to be identified but will likely involve circadian disruption. (C) Prospective risks of irregular sleep: most studies to date remain cross-sectional. Dose–response relationships will have to be established in longitudinal studies.

We still understand remarkably little about which factors lead people to experience irregular sleep patterns. It seems evident that irregular sleep patterns must arise due to some combination of:

- Imposed (environmental) factors, such as work schedules or family obligations [6], as well as environmental factors disruptive to sleep, such as ambient temperature, noise, or lighting (e.g. hospital settings [7]).
- Endogenous factors or traits, such as an individual’s sensitivity to sleep loss or properties of the circadian clock that affect the timing and stability of circadian rhythms, such as intrinsic circadian period or sensitivity to light [8–10].
- Behavioral choices that an individual makes, such as when they exercise or consume their meals, or alter their sleep for leisure and social activities [11] (Figure 2, A).

All three factors will be subject to changes across the life course, including developmental changes in circadian biology, such as circadian phase delay in adolescence and young adulthood [12, 13], altered circadian and homeostatic functioning in older individuals [14], and age-related changes in circadian photoreception [15]. Currently, we do not know the relative importance of these factors, nor do we understand how these factors interact. In real-world scenarios, interactions are likely. For instance, many work schedules lead to curtailed sleep on workdays and subsequent catch-up sleep on days off, resulting in irregular sleep [16]. However, an individual’s sleep–wake response to this work pattern could depend on all three of the above factors. Furthermore, shift work is known to affect dietary decisions [17], exercise [18], and light exposure [19], all of which can influence sleep–wake behavior [20–22]. Meanwhile, an individual’s circadian physiology may also influence how their sleep–wake patterns respond to shift work [23–25]. Studies to tease these factors and their interactions apart are needed to understand how we can effectively improve sleep regularity at a population level. There are many possible approaches to addressing this research gap, beyond observational or behavioral studies, including computational modeling to investigate mechanisms and genome-wide interaction studies to determine how genetic or physiological traits interact with environmental factors. Additionally, it will be important to study these factors in a variety of populations (e.g. shift workers, clinical populations, children), since the relative importance of the drivers of irregular sleep will likely differ between populations. For instance, endogenous factors may play a dominant role in generating irregular sleep in certain clinical populations, whereas the imposed work schedule may play a dominant role in generating irregular sleep in a shift worker. Determining the specific contribution of shift schedules to sleep regularity may help to guide the design and selection of healthier schedules.

### Key questions

- How do we disentangle environmental, physiological, and behavioral factors that contribute to irregular sleep?
- How do these factors differentially affect sleep regularity in different populations and life stages?
- If regular sleep is important for health, what does this mean for permanent vs. rotational shift schedules?

## Determining the Prospective Risks of Irregular Sleep

Irregular sleep has now been associated with a wide range of adverse health outcomes [2, 3, 26, 27]. Yet, we do not know the degree to which these relationships are causal, as most studies have been cross-sectional. In certain cases, we believe that irregular sleep is likely to be a primary cause of poor health. In other cases, irregular sleep may be a marker of poor health or a downstream contributor to poor health; in these cases, sleep regularity would remain a valuable biomarker but may not be the ideal target for intervention. Prospective studies of the effects of irregularity on health are needed to establish when and to what degree these relationships are causal.

Secondary to establishing a causal relationship, we need to better map the dose–response relationship between irregular sleep and adverse outcomes (Figure 2, C). Specifically, we need to identify what is a healthy level of sleep regularity [28, 29], while appreciating that this may differ based on the outcome and individual characteristics including developmental stage. As is the case for sleep duration, what is regarded as an optimal, healthy level of sleep regularity may differ across the lifespan. Furthermore, an alarm clock may help to maintain regular sleep patterns, via consistent wake-up times between days, but it may alternatively promote irregular sleep patterns if used variably between days (e.g. weekdays vs. weekends). Accordingly, sleep regularity must be weighed against competing sleep demands, including “catch-up sleep” in individuals who are acutely or chronically sleep restricted [30]. As has been done for sleep duration [31], these limits will likely have to be established by triangulation across a range of outcomes to ultimately establish what is a healthy range.

### Key questions

- What level of irregular sleep over what period can be tolerated without adverse consequences?
- If irregular sleep patterns increase risks, how long does it take to return to baseline and what are safe and effective strategies to accelerate the return?
- Which health outcomes are directly caused by irregular sleep patterns?
- Is it better to catch up on lost sleep, at the expense of irregular sleep times, or to maintain a regular sleep schedule, at the expense of accruing sleep debt? Is the alarm clock an anchor or an irregularity enabler?

## Identifying the Physiological Mechanisms by which Irregular Sleep Impacts Health

Arguably, the greatest gap in our knowledge pertains to the specific pathways by which irregular sleep leads to adverse health outcomes. A credible hypothesis is that irregular sleep leads to a disruption of circadian rhythms, since people with irregular sleep will tend to have less regular environmental time cues (“zeitgebers”), including light/dark exposure [1, 4], eating, and exercising (Figure 2, A and B). Given the circadian system’s ubiquitous role in regulating our biology [32], circadian disruption would explain the wide range of detrimental outcomes associated with irregular sleep. Candidate physiological mechanisms linking circadian disruption to negative health outcomes include impaired glucose tolerance and insulin sensitivity, impaired immune function, elevated blood pressure and reduced heart rate variability, and disrupted gut microbiome rhythms [32–35]. Circadian disruption can arise at multiple physiological levels [33–35], meaning some rhythms may be mistimed with the external 24-h day (e.g. sleeping at different times each day), while other rhythms remain aligned (e.g. regular eating habits). We need to tease these pathways apart to discover the causal mechanisms that link irregular sleep to poor health. This could involve a combination of experimental and observational approaches, investigating the multi-dimensional nature of irregularity and determining how these dimensions map to outcomes. For instance, mental health outcomes may be particularly sensitive to the regularity of light exposure patterns [36, 37].

Establishing causal mechanisms for sleep regularity and health ultimately depends on the use of suitable metrics to assess sleep regularity. The development of sleep regularity metrics remains an area of active research. Recently proposed metrics that assess day-to-day variability in sleep patterns, such as the Sleep Regularity Index [38] and Composite Phase Deviation metric [39], provide complementary value to other metrics that have traditionally been used to measure variability in sleep patterns (e.g. standard deviation of sleep onset time). Combining two or more sleep regularity metrics that quantify different aspects of regularity may therefore provide insights into which specific aspects of irregularity link to health risks [40]. Furthermore, sleep regularity metrics could be generalized to simultaneously measure regularity across multiple aspects of behavior (e.g. light [4], meal timing [41]). Ultimately, this approach will lay the groundwork for designing the most effective interventions based on regularity.

### Key questions

- Is it better to keep behavioral rhythms in sync with one another, or to strive for regularity in whichever dimension one can (e.g. in cases where some dimensions cannot be controlled, such as in shift work)?
- Which input signals (e.g. light, meal timing) and which physiological pathways causally link irregular sleep to poor health outcomes?
- What is the best approach to quantifying regularity to answer these questions [40]?

## Conclusion

Recent discoveries have put the spotlight on sleep regularity as a key dimension of health. As more investigators work to study the importance of sleep regularity for a myriad of outcomes, now is the critical time to steer our collective efforts. Even as sleep regularity enthusiasts, we must acknowledge that critical gaps remain in our understanding. In our view, the three most critical of these are: (1) What are the factors that drive irregular sleep? (2) How does sleep regularity relate prospectively to outcomes? and (3) What are the physiological mechanisms by which irregular sleep impacts health? By addressing these questions, we will create a strong theoretical basis for the field and advance our understanding of how irregular sleep leads to adverse outcomes. Only then can we translate research findings into effective, pinpointed interventions to improve human health.

## Data Availability

No new data were generated or analyzed in support of this research.
